# SP2‐induced circPUM1 modulates chemoresistance and nature killer cell toxicity in oral squamous cell carcinoma

**DOI:** 10.1111/jcmm.17888

**Published:** 2023-08-09

**Authors:** Lian Liu, Chen Zou, Xiaozhi Lv, Haigang Wei, Siyuan Wu, Jing Song, Zhe Tang, Hailing Luo, Xia Li, Yilong Ai

**Affiliations:** ^1^ Foshan Stomatological Hospital, School of Medicine Foshan University Foshan China; ^2^ Department of Oral and Maxillofacial Surgery, Zhujiang Hospital Southern Medical University Guangzhou China

**Keywords:** chemoresistance, circPUM1, miR‐770‐5p, NAP1L1, nature killer cell, oral squamous cell carcinoma, SP2

## Abstract

Oral squamous cell carcinoma (OSCC) is a type of tumour found in the cavity that is characterized by differentiation and metastasis to the lymph nodes. Although diagnosis strategy and clinical treatment have recently improved, the outcomes for OSCC patients remain unsatisfactory. This study verified the characteristics of circPUM1 in OSCC cells, subsequently generating dysregulated circPUM1 cell models, showing that circPUM1 promoted chemoresistance and natural killer (NK) cell toxicity. Furthermore, the transcription factor SP2 regulated the expression of circPUM1 in OSCC cells, circPUM1 acted as a molecular sponge for miR‐770‐5p. Moreover, Nucleosome Assembly Protein 1 Like 1 (NAP1L1) is a downstream target for miR‐770‐5p and essential for circPUM1‐mediated cisplatin resistance and NK cell cytotoxicity in OSCC cells. The network composed of SP2, circPUM1, miR‐770‐5p and NAP1L1 in OSCC appears to be a promising avenue for the development of novel targets for diagnosing or treating OSCC.

## 1 INTRODUCTION

Oral squamous cell carcinoma (OSCC) is a type of tumour found in the cavity and is well known for its features of differentiation and metastasis to the lymph nodes.^1^ Globally, nearly 540,000 patients were newly diagnosed with OSCC, and the mortality from OSCC ranks the 15th among all malignancies.^2^ Despite the considerable improvement in the diagnosis and therapy strategies of OSCC recently, the overall and 5‐year survival rate remain unsatisfactory.^3^, ^4^, ^5^ The risk factors for OSCC developing into metastatic status, pathological differentiation, radio‐resistance and chemoresistance are challenging for clinical interventions.^6^, ^7^ It is essential to conduct further studies in order to develop novel diagnostic and therapeutic targets for OSCC.

Circular RNAs (circRNAs) are newly classified as endogenous non‐coding RNAs and characterized by abundant, conserved and stable features in eukaryotic cells.^8^, ^9^ In the past two decades, the introduction of high‐throughput sequencing technology has sparked a surge of scientific interest in understanding the molecular structure and functional significance of circRNAs. Several studies have reported on the biological functions of multiple circRNAs in various cancers, including glioma, liver cancer, bladder cancer, renal cell carcinoma, prostate cancer, gastric cancer and pancreatic cancer, providing accumulating evidence of their potential role in cancer biology.^10^, ^11^, ^12^, ^13^, ^14^, ^15^, ^16^ In OSCC, circUHRF1 had been demonstrated as one oncogene in OSCC progression,^17^ and by mediating the EMT and the PI3K/Akt/mTOR pathway through the circRNA/miRNA/mRNA network, circEPSTI1 accelerates OSCC progression.^18^ CircIGHG promotes OSCC progression via sponging miR‐142‐5p and modulating IGF2BP3 expression.^19^ Thus, circRNAs have key roles in OSCC initiation or progression.

Circular RNA Pumilio RNA Binding Family Member 1 (circPUM1), also known as has_circ_0000043, is back‐spliced from the PUM1 gene. CircPUM1 is dysregulated and possesses functions in multiple cancers, for instance, ovarian cancer, lung cancer, hepatocellular cancer, papillary thyroid cancer, pancreatic cancer and renal cell carcinoma,^20^, ^21^, ^22^, ^23^, ^24^ but it is unclear whether circPUM1 is involved in OSCC progression.

The aim of this study was to investigate the biological role of circPUM1 in the progression of OSCC. Initially, the circular RNA features of circPUM1 were elucidated in OSCC cells. Following this, the functional role of circPUM1 in NK cell‐mediated toxicity and cisplatin chemoresistance in OSCC cells was explored. CircPUM1 aggravated chemoresistance and NK cell toxicity and was transcriptionally regulated by SP2, with the miR‐770‐5p/NAP1L1 axis essential for circPUM1‐mediated NK cell toxicity and cisplatin chemoresistance.

## 2 MATERIALS AND METHODS

### 2.1 Cell treatment

The American Type Culture Collection (ATCC) provided the cell lines used in this study, including HSC‐6, SCC‐9, UM2, UM1, CAL‐27, HSC‐3, SCC‐1, SCC‐15 and human oral keratinocyte (HOK). These cell lines were cultured in DMEM supplemented with 10% FBS (Gibco) in a 5% CO_2_ environment at 37°C. GenePharma (Shanghai, China) synthesized the shRNAs, lentiviruses, small interfering RNAs, mimics and inhibitors. Transfections were performed using the Lipofectamine 2000 kit (Life Technologies), and all experiments were performed in triplicate.

### 2.2 RNase R and actinomycin D treatment assay

RNA was extracted from the cells and treated with RNase (Geneseed Biotech, China) for 30 min. Additionally, HSC‐6 and SCC‐9 cells were exposed to 2 μg/mL actinomycin D (Medchemexpress, USA) for 24 h.

### 2.3 Reverse transcription and quantitative PCR (RT‐qPCR) assay

To extract RNA from the cells, an RNAiso Plus kit (TaKaRa, Japan) was used, followed by reverse transcription using the PrimeScript RT reagent kit (TaKaRa, Japan). SYBR Premix Ex Taq (TaKaRa) was used for qRT‐PCR, with GAPDH and U6 serving as internal controls. The miRNA expression was quantified using an miRNA‐X PCR assay kit (Clontech, USA), and the relative RNA expression level was evaluated using the $2^{-\Delta \Delta C_{t}}$ method. The following primers were used: circPUM1; F: 5′‐CCAAGCCTGTGGAGGATTTCT‐3′, R: 5′‐CACATCACCCTCCTCCTTCAA‐3′; PUM1; F: 5′‐GGTCCAGAAGATGATTGAC‐3′, R: 5′‐ TACGAAGAGTTGCGATGTGG‐3′; SP2; F: 5′‐CGTAGAATTCCAAGCGATCCACAGATGAGCATG‐3′, R: 5′‐GATCGTCGACAGTTGGCCTTACAAGCCCTTC‐3′; miR‐770‐5p; F: 5′‐AGCACCACGTGTCTGG‐3′, R: 5′‐GAACATGTCTGCGTATCTC‐3′; NAP1L1; F: 5′‐TCCTGAAGTTCCTGAGAGTG ‐3′, R: 5′‐CACATACATCCTGCTTCACTG‐3′; GADPH; F: 5′‐CCCACATGGCCTCCAAGGAGTA‐3′, R: 5′‐GTGTACATGGCAACTGTGAGGAGG‐3′; U6; F: 5′‐TTCACGAATTTGCGTGTCAT‐3′, R: 5′‐CGCTCGGCAGCACATATAC‐3′. Experiments were repeated three times.

### 2.4 Western blot assay

To extract the proteins from the transfected cells, RIPA buffer (Beyotime) was used for cell lysis. The proteins were then separated by SDS‐PAGE and transferred to a PVDF membrane. The membrane was blocked in non‐fat milk for 1 h before being incubated with primary antibodies, followed by secondary antibodies. The protein bands were visualized using an ECL Prime Western Blotting Kit (Beyotime).

### 2.5 Cell viability detection

To determine cell viability, a CCK‐8 assay kit (Beyotime) was used. Treated cells were seeded in a 96‐well plate at a density of 10^4^ cells per well with fresh medium for 12 h, followed by the addition of cisplatin (CDDP) at indicated time points. After incubating with the CCK‐8 solution for 120 min, the absorbance was measured at 450 nm using a microplate reader (BioTek).

### 2.6 Measurement of NK cell‐mediated toxicity

NK cell‐mediated toxicity was measured by the calcein release assay, perforin polarization assay and conjugation assay, as previously described.^25^

### 2.7 Bioinformatics analysis

NCBI (https://www.ncbi.nlm.nih.gov/), UCSC (http://genome.ucsc.edu/) and JASPAR (http://jaspar.genereg.net/) datasets were used to identify putative transcriptional regulators of circPUM1. MiRNA targets for circPUM1 were then identified using Starbase (http://starbase.sysu.edu.cn/), with a CLIP Data strict stringency ≥5 and class ≥8 mer. The DIANA tool (microT: http://diana.imis.athena‐innovation.gr/), miRmap (https://mirmap.ezlab.org/), PITA (http://genie.weizmann.ac.il/pubs/mir07/mir07_dyn_data.html) and PicTar (http://www.pictar.org/), with CLIP Data strict stringency ≥5, and Degradome Data low stringency ≥1 were used to identify potential mRNA targets for miR‐770‐5p.

### 2.8 ChIP assay

The ChIP assay was performed using the MagnaChIP Kit (Millipore), anti‐IgG and anti‐SP2 (Sigma) and analysed by qRT‐PCR.

### 2.9 RNA immunoprecipitation (RIP)

Approximately, 20 μL of proteins from the treated OSCC cells were collected and used as the input sample. The proteins were incubated with magnetic beads on ice. After the RIP assay, the RNAs were purified, dissolved in the diethylpyrocarbonate‐treated water and then quantified via qRT‐PCR.

### 2.10 Luciferase reporter gene assay

HSC‐6 and SCC‐9 cells were co‐transfected with miR‐770‐5p mimics or controls and luciferase reporter vectors containing wild‐type (WT) or mutant‐type (MUT) circPUM1 or NAP1L1 using Lipofectamine 2000. The luciferase intensity was measured using a dual luciferase reporter assay system (Promega).

### 2.11 Statistical analysis

The statistical analysis was performed using SPSS 16.0v from IBM and the results were presented as mean ± SD. The difference between groups was analysed using Student's *t*‐test or one‐way anova, and a *p*‐value of <0.05 was statistically significant.

## 3 RESULTS

### 3.1 Characterizations of circPUM1 in OSCC cells

**FIGURE. 1:**
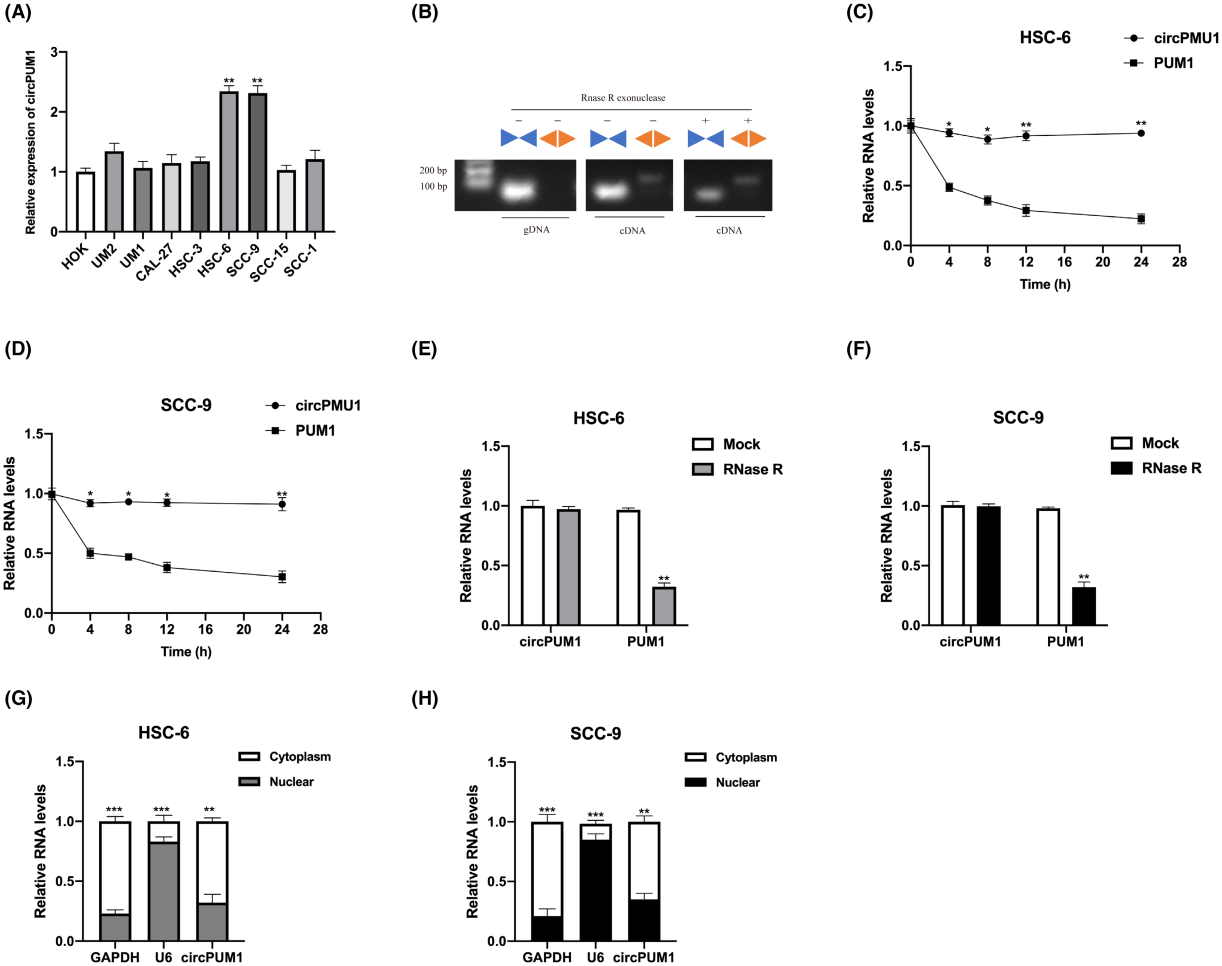
Expression of circPUM1 in OSCC cells. (A) Expression of circPUM1 in OSCC cell lines and HOK cells was detected by qRT‐PCR. (B) Divergent and convergent primers were used to amplify circPUM1 and mPUM1 from gDNA, cDNA without RNase R treatment before RT‐PCR and cDNA with RNase R treatment before RT‐PCR in HSC‐6 cell, respectively. (C, D) The expression of circPUM1 and PUM1 in HSC‐6 and SCC‐9 after actinomycin D treatment was detected by qRT‐PCR. (E, F) The expression stability of circPUM1 and PUM1 in HSC‐6 and SCC‐9 cells after RNase R treatment quantified by qRT‐PCR. (G, H) The distribution of circPUM1 in HSC‐6 and SCC‐9 cells was measured by a cellular fragment assay. Data presented as mean ± SD, *p < 0.05, **p < 0.01, ***p < 0.001.

Abundant expression of CircPUM1 was observed in OSCC cells, particularly in HSC‐6 and SCC‐9 cells (Figure 1A). The amplification of the PCR product by divergent primers specific to CircPUM1 (Figure 1B) was consistent with its circular form. Resistance to RNase R digestion was expected and confirmed when total RNA was treated with RNase R prior to PCR amplification. Moreover, in HSC‐6 and SCC‐9 cells, CircPUM1 was found to be markedly more stable than linear RNA PUM1 after treatment with actinomycin D (Figure 1C,D) or RNase R digestion (Figure 1E,F). The cytoplasm of HSC‐6 and SCC‐9 cells showed the predominant expression of CircPUM1 (Figure 1G,H). These findings suggest that CircPUM1 may play a functional role in OSCC cells.

### 3.2 CircPUM1 dysregulation influences chemoresistance to cisplatin and nature killer cell susceptibility to OSCC cells

**FIGURE. 2:**
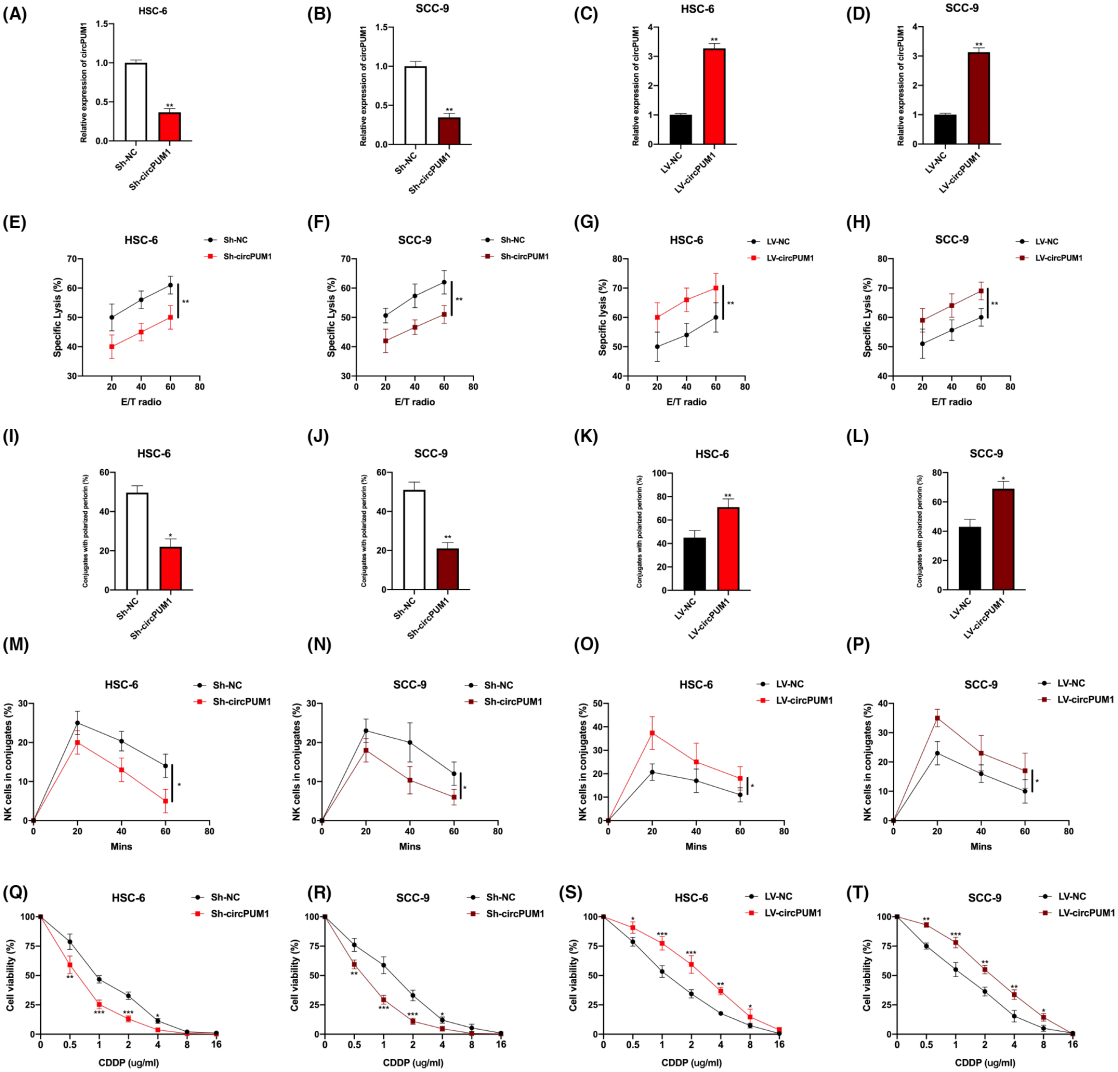
CircPUM1 dysregulation influences cisplatin chemoresistance and NK cell susceptibility in OSCC cells. (A–D) CircPUM1 dysregulation cell models were generated by infecting Sh‐NC, Sh‐circPUM1, LV‐NC and LV‐circPUM1 into HSC‐6 and SCC‐9 cells, as indicated. (E–H) Calcein release assay to evaluate NK cell toxicity in infected HSC‐6 and SCC‐9 cells. (I–L) Perforin polarization assay to assess NK cell toxicity in infected HSC‐6 and SCC‐9 cells. (M–P) Conjugation assay to assess the conjugation status of NK and OSCC cells. (Q–T) The cisplatin (CDDP) chemoresistance status in circPUM1 dysregulated HSC‐6 and SCC‐9 cells was measured by the cell viability assay. Data presented as mean ± SD, *p < 0.05, **p < 0.01, ***p < 0.001.

NK cells are innate lymphocytes that have a short half‐life and linear differentiation. Recent studies suggest that NK cells can have a significant impact on the progression of human cancer and can even lead to prolonged patient survival,^26^, ^27^, ^28^, ^29^, ^30^ but whether circPUM1 influences NK cell susceptibility in OSCC cells remains unclear. Up‐ or downregulated circPUM1 OSCC cell models were generated (Figure 2A–D) for functional investigation. The calcein release assay (Figure 2E–H), perforin polarization assay (Figure 2I–L) and conjugation assay (Figure 2M–P) revealed that OSCC cell susceptibility towards NK cells decreased upon circPUM1 overexpression but increased upon circPUM1 downregulation. Subsequently, we also investigated the effect of circPUM1 on OSCC cells upon CDDP treatment. CDDP‐resistance cell models were constructed as shown in Figure S1. CircPUM1 knockdown markedly decreased cell viability for HSC‐6 and SCC‐9 cells, but this was increased by circPUM1 overexpression (Figure 2Q–T), indicating that circPUM1 influences the therapeutic efficiency of CDDP on OSCC cells.

### 3.3 SP2 transcriptionally regulates circPUM1 expression in OSCC cells

**FIGURE. 3:**
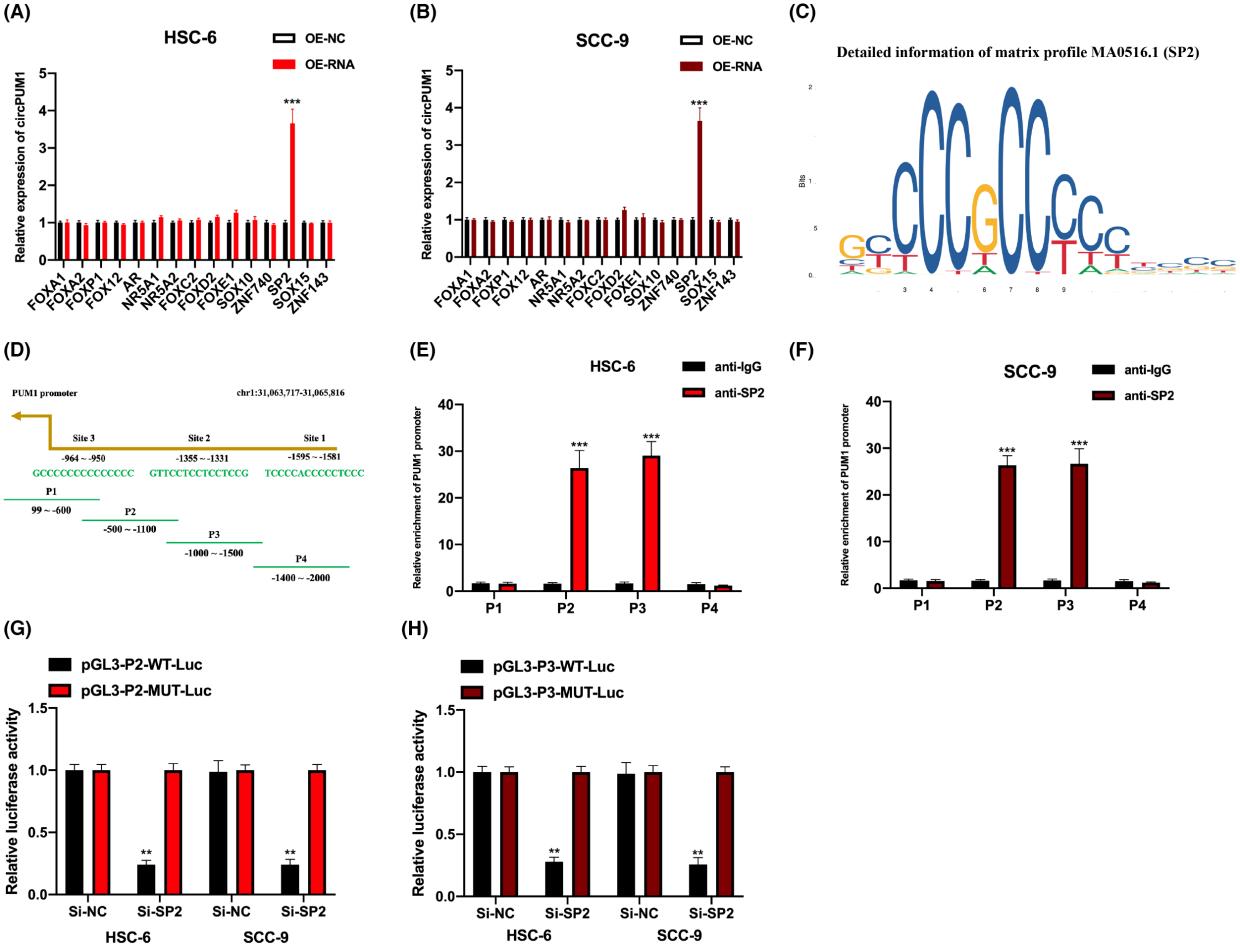
SP2 transcriptionally regulates circPUM1 expression in OSCC cells. (A, B) HSC‐6 and SCC‐9 cells were infected with the indicated siRNAs, and the relative expression of circPUM1 was measured by qRT‐PCR. (C) The binding motif of SP2 was obtained from the JASPAR database. (D) The predicted binding sequences of the PUM1 promoter to SP2. (E, F) ChIP‐anti‐SP2 assay to evaluate the relationship between the putative binding regions of PUM1 to SP2, in HSC‐6 and SCC‐9 cells. (G, H) Luciferase assay to demonstrate the correlation between SP2 and the P2/P3 region in the PUM1 promoter in HSC‐6 and SCC‐9 cells. Data presented as mean ± SD, **p < 0.01, ***p < 0.001.

The upstream factors regulating circPUM1 expression in OSCC cells were explored. Fifteen putative regulators of circPUM1 expression in OSCC cells were identified through bioinformatics analysis. Upon overexpression of SP2, circPUM1 expression was found to be significantly upregulated in HSC‐6 and SCC‐9 cells, suggesting that regulation of circPUM1 expression may be attributed to SP2 (Figure 3A,B). The predicted binding motif of SP2 and putative binding regions in the PUM1 promoter were obtained from the JASPAR database (Figure 3C,D). The results of the ChIP assay showed that anti‐SP2 complexes were abundantly enriched in the P2 and P3 regions of the PUM1 promoter, but not anti‐IgG (Figure 3E,F), indicating a possible binding of SP2 with the P2 and P3 regions of the PUM1 promoter. Furthermore, SP2 was found to induce circPUM1 expression in OSCC cells (Figure 3G,H).

### 3.4 CircPUM1 sponges miR‐770‐5p

**FIGURE. 4:**
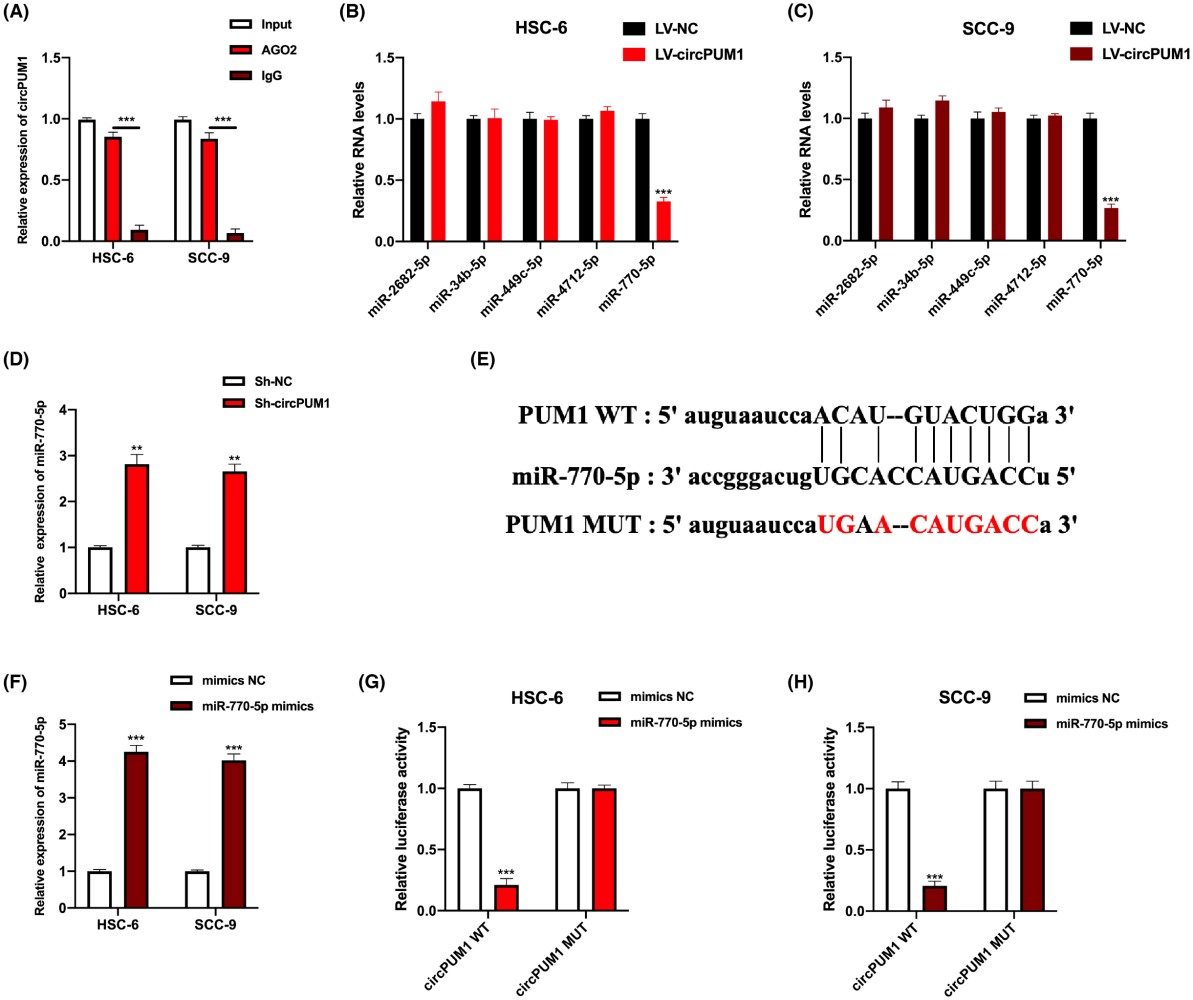
CircPUM1 sponges miR‐770‐5p. (A, B) The potential binding ability of circPUM1 to AGO2 (the core components of mRNA RISC) was measured by the AGO‐2‐RIP assay. (C, D) Relative expression of putative miRNAs in circPUM1 down‐ or upregulated HSC‐6 and SCC‐9 cells measured by qRT‐PCR. (E) The predicted binding motif between circPUM1 and miR‐770‐5p. (F) HSC‐6 and SCC‐9 cells were infected with miR‐770‐5p mimics and controls and then transfection efficiency was assessed by qRT‐PCR. (G, H) HSC‐6 and SCC‐9 cells were stably transfected with the luciferase‐constructed vectors and miR‐770‐5p mimics as indicated and the luciferase activity was detected. Data shown as mean ± SD, **p < 0.01, ***p < 0.001.

Next, we investigated the downstream factors of circPUM1, showing that circPUM1 was efficiently enriched in anti‐AGO2 compared to anti‐IgG (Figure 4A), suggesting that circPUM1 can bind miRNAs. The bioinformatics analysis predicted five potential miRNA targets for circPUM1. MiR‐770‐5p expression was markedly regulated on circPUM1 dysregulation in HSC‐6 and SCC‐9 cells (Figure 4B–D). The sequence analysis showed that circPUM1 shared miRNA response elements (MREs) with miR‐770‐5p (Figure 4E). Subsequently, miR‐770‐5p overexpressed cell models were generated, by stably transfecting miR‐770‐5p mimics and controls into HSC‐6 and SCC‐9 cells (Figure 4F). The correlation between circPUM1 and miR‐770‐5p in HSC‐6 and SCC‐9 cells was assessed and further confirmed by a luciferase assay (Figure 4G,H).

### 3.5 MiR‐770‐5p modulates chemoresistance to cisplatin and nature killer cell toxicity to OSCC cells

**FIGURE. 5:**
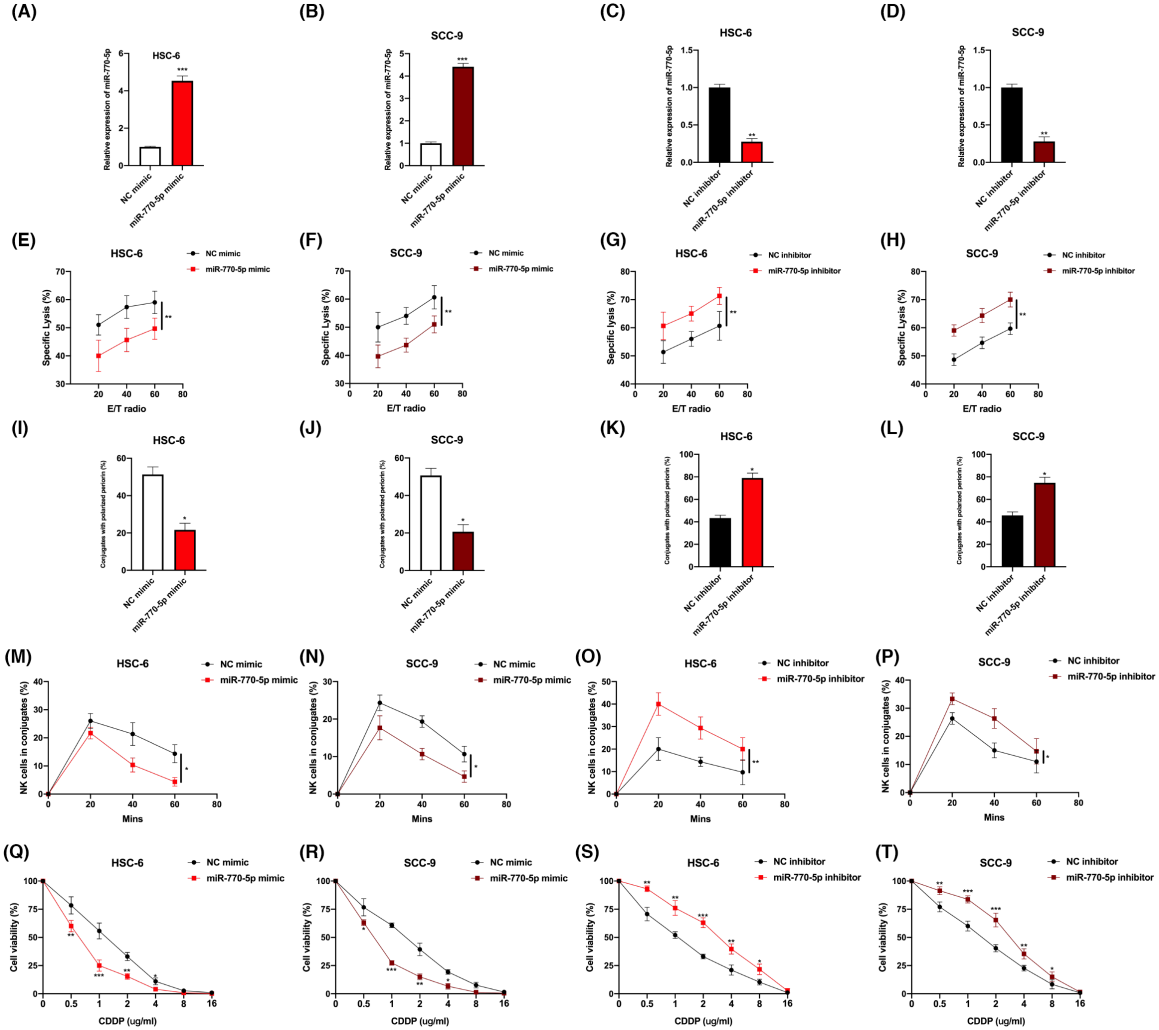
MiR‐770‐5p modulates cisplatin chemoresistance and NK cell susceptibility in OSCC cells. (A–D) HSC‐6 and SCC‐9 were stably infected with NC mimics, miR‐770‐5p mimics, NC inhibitors and miR‐770‐5p inhibitors, as indicated. Relative expression of miR‐770‐5p was measured by qRT‐PCR. (E–H) NK cell toxicity in infected HSC‐6 and SCC‐9 cells was assessed by the calcein release assay. (I–L) Numbers of NK cells containing perforin were calculated to evaluate NK cell toxicity in infected HSC‐6 and SCC‐9 cells. (M–P) Conjugation assay to measure the conjugation status between NK cells and OSCC cells. (Q–T) The cisplatin (CDDP) chemoresistance status in circPUM1 dysregulated HSC‐6 and SCC‐9 cells was evaluated by the cell viability assay. Data shown as mean ± SD, *p < 0.05, **p < 0.01, ***p < 0.001.

Cell models with up‐ and down‐regulated miR‐770‐5p were generated in HSC‐6 and SCC‐9 cells by infecting miR‐770‐5p mimics, inhibitors and their respective normal controls, as shown in Figure 5A–D. The objective was to investigate the influence of miR‐770‐5p dysregulation on NK cell toxicity. The results of the calcein release assay (Figure 5E–H), perforin polarization assay (Figure 5I–L) and conjugation assay (Figure 5M–P) showed that overexpression of miR‐770‐5p promoted OSCC cell susceptibility to NK cells, which was attenuated by miR‐770‐5p downregulation. Additionally, the results of cell viability assays (Figure 5Q–T) indicated that miR‐770‐5p has a functional role in OSCC cells, as overexpression of miR‐770‐5p inhibited cell viability, while downregulation of miR‐770‐5p promoted cell viability.

### 3.6 MiR‐770‐5p directly targets NAP1L1

**FIGURE. 6:**
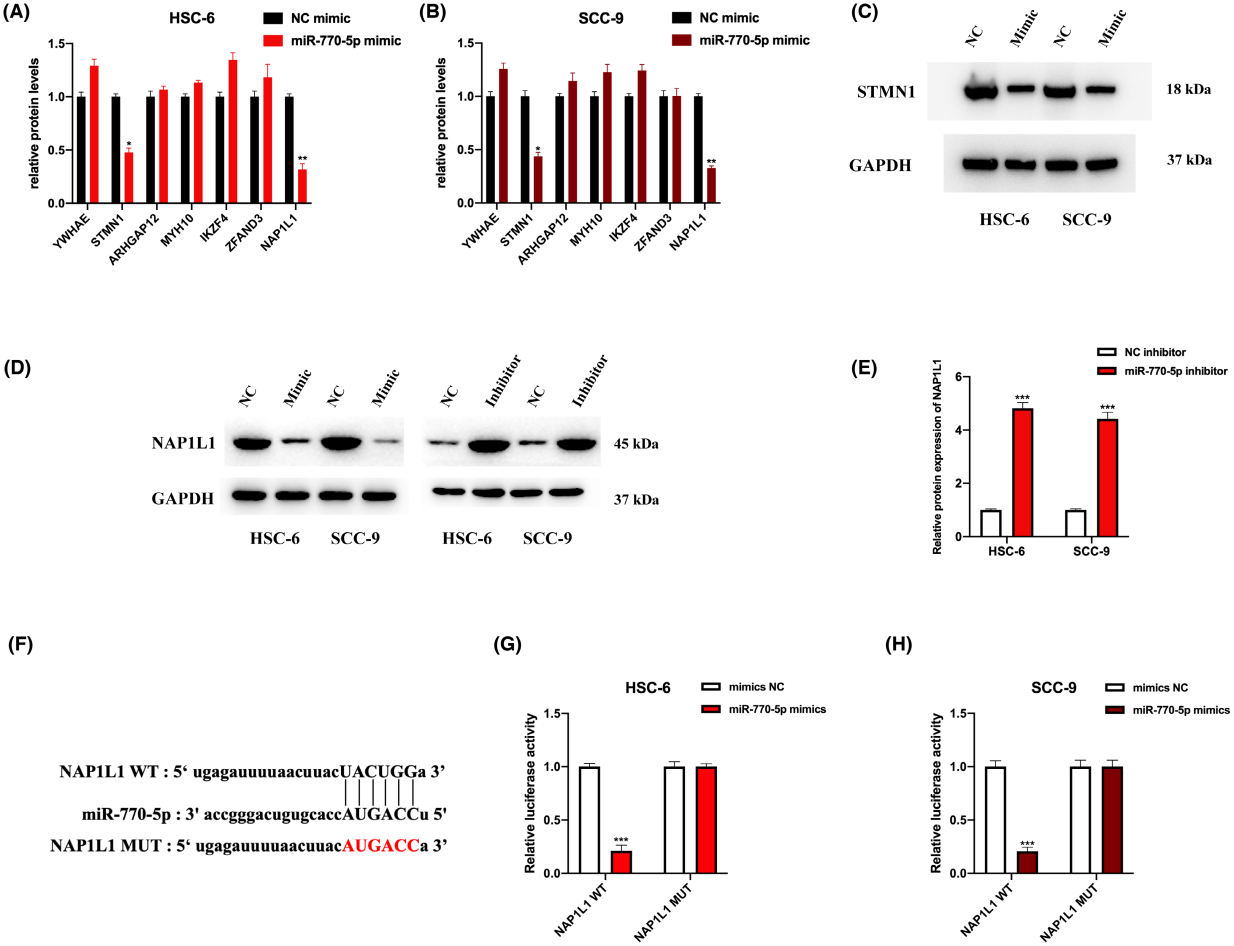
MiR‐770‐5p directly targets NAP1L1. (A, B) Relative expression of putative targets in miR‐770‐5p overexpressed HSC‐6 and SCC‐9 cells were measured by Western blot, and results were statistically analysed. (C) Protein expression of STMN1 in miR‐770‐5p overexpressed HSC‐6 and SCC‐9 cells was detected by western blot. (D) Protein expression of NAP1L1 in miR‐770‐5p overexpressed or downregulated HSC‐6 and SCC‐9 cells were detected by Western blot. (E) Statistical analysis of the protein expression of NAP1L1 in miR‐770‐5p downregulated HSC‐6 and SCC‐9 cells. (F) The predicted binding motif between miR‐770‐5p and NAP1L1. (G, H) The potential correlation between miR‐770‐5p and NAP1L1 was assessed by the luciferase assay in HSC‐6 and SCC‐9 cells. Data shown as mean ± SD, *p < 0.05, **p < 0.01, ***p < 0.001.

The bioinformatics analysis indicated seven putative mRNA downstream targets for miR‐770‐5p. Nucleosome assembly protein 1‐like 1 protein (NAP1L1) and stathmin 1 (STMN1) were markedly dysregulated in HSC‐6 and SCC‐9 cells after transfection with the miR‐770‐5p mimic or inhibitor, respectively (Figure 6A–C), the relationship between miR‐770‐5p and STMN1 has been verified by a previous study.^31^ In addition, MREs of miR‐770‐5p were identified in the sequence analysis of NAP1L1 (Figure 6D). The luciferase activity assay showed that the miR‐770‐3p mimic significantly inhibited the activity of the wild‐type sequence of NAP1L1, but not the mutant type in OSCC cells (Figure 6E,F). Furthermore, the expression of NAP1L1 was negatively regulated by miR‐770‐5p in OSCC cells (Figure 6G), indicating a direct targeting of NAP1L1 by miR‐770‐5p and a negative regulation of NAP1L1 expression in OSCC cells.

### 3.7 NAP1L1 is essential for the functional role of circPUM1 in OSCC cells

**FIGURE. 7:**
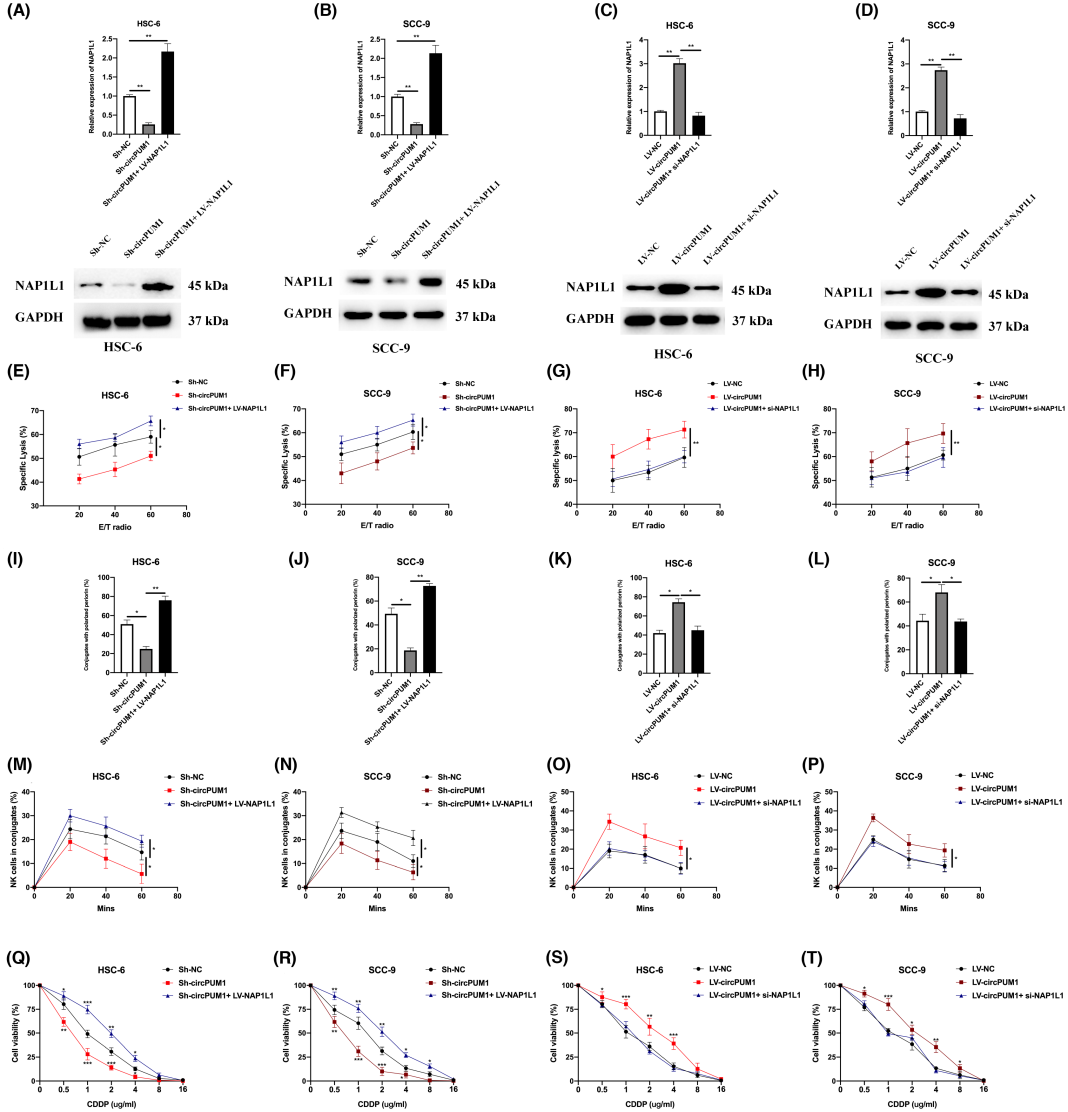
NAP1L1 is essential for the functional role of circPUM1 in OSCC cells. (A–D) HSC and SCC‐9 cells were stably transfected with Sh‐NC, Sh‐circPUM1, Sh‐circPUM1 + LV‐NAP1L1, LV‐NC, LV‐circPUM1 and LV‐circPUM1 + si‐NAP1L1 as indicated, and NAP1L1 expression was measured by qRT‐PCR and western blotting. (E–H) NK cell toxicity in infected HSC‐6 and SCC‐9 cells was assessed by the calcein release assay. (I–L) Numbers of NK cells containing perforin were calculated to assess cell toxicity in infected HSC‐6 and SCC‐9 cells. (M–P) Conjugation assay was used to measure the conjugation status between NK cells and OSCC cells. (Q–T) The cisplatin (CDDP) chemoresistance status in infected HSC‐6 and SCC‐9 cells was evaluated by the cell viability assay. Data presented as mean ± SD, *p < 0.05, **p < 0.01, ***p < 0.001.

In this study, the role of NAP1L1 in circPUM1‐mediated chemoresistance and NK cell toxicity in OSCC cells was investigated. To accomplish this, NAP1L1 was overexpressed in circPUM1 downregulated cells and its expression was silenced in circPUM1 upregulated cells in HSC‐6 and SCC‐9 cells (Figure 7A–D). The effect of NAP1L1 expression on NK cell toxicity was determined using calcein release assay (Figure 7E–H), perforin polarization assay (Figure 7I–L) and conjugation assay (Figure 7M–P). The results demonstrated that NAP1L1 overexpression markedly reversed downregulated‐circPUM1‐mediated attenuation of NK cell toxicity, and NAP1L1 downregulation significantly rescued upregulated‐circPUM1‐mediated aggravation of NK cell toxicity. Furthermore, NAP1L1 overexpression promoted cell viability reduced by downregulated circPUM1, and NAP1L1 downregulation alleviated the cell viability promoted by upregulated circPUM1 in CDDP‐treated OSCC cells (Figure 7Q–T). These findings suggest that NAP1L1 plays a crucial role in circPUM1‐mediated chemoresistance and NK cell toxicity towards OSCC cells.

## 4 DISCUSSION

NK cells are one class of peripheral‐blood lymphocytes that play an essential role in the innate immune system response to viruses and tumours,^32^ in particular, the immune escape of tumour cells.^33^ Thus, their potential in cell therapy development is not ignorable. Previous studies have reported that circRNAs play a critical role in NK cell dysfunctions. In hepatocellular carcinoma, circUHRF1 exerts its role in NK cell exhaustion, and hsa_circ_0007456 regulates NK cell‐mediated cytotoxicity.^25^, ^34^ The role of circPUM1 in NK cell behaviours has not been previously investigated. Our study showed that circPUM1 promoted NK cell cytotoxicity in OSCC cells, which enriched the biological profile of circPUM1.

Emerging evidence suggests that the expression of circRNA could be regulated by transcription factors.^35^, ^36^ The regulation of circPUM1 expression in OSCC cells by the transcriptional regulator SP2 was investigated to gain a deeper understanding of the underlying molecular mechanisms of circPUM1 in OSCC. It was discovered that SP2 can modulate the expression of circPUM1, thus broadening the knowledge of the molecular role of circPUM1.

Cisplatin is widely applied in the clinical treatment of OSCC, normally combined with 5FU (5‐fluorouracil), and docetaxel (TPF).^37^ However, the emergence of cisplatin chemoresistance in OSCC patients has necessitated the investigation of the underlying molecular mechanisms of cisplatin resistance. The present study revealed that circPUM1 dysregulation influences cisplatin chemoresistance in OSCC cells, with circPUM1 overexpression increasing CDDP‐treated OSCC cell viability, and circPUM1 downregulation having the opposite effect. These results suggest that circPUM1 might be a novel target for cisplatin resistance research in OSCC.

The molecular analyses revealed that circPUM1 acted as a molecular sponge for miR‐770‐5p in OSCC cells and regulated miR‐770‐5p expression in OSCC cells. Although there are many studies exploring the functions of miR‐770‐5p in various diseases, including ovarian cancer, glioblastoma, breast cancer and diabetic nephropathy,^38^, ^39^, ^40^, ^41^ our study firstly verified the biological role of miR‐770‐5p in OSCC. Furthermore, we also identified its downstream molecular, NAP1L1, which also remains uncovered in OSCC.

It has been well documented that NAP1L1 plays essential roles in multiple cancers^42^, ^43^, ^44^ and participates in chemoresistance for hepatocellular carcinoma and glioma.^45^, ^46^ Interestingly, neither the biological nor mechanical role of NAP1L1 in OSCC has been investigated. Our research identified that NAP1L1 is a downstream target for miR‐770‐5p, and essential for circPUM1‐mediated chemoresistance and NK cell toxicity towards OSCC cells.

Despite our study having partially demonstrated the role of circPUM1 in OSCC and revealing an SP2/circPUM1/miR‐770‐5p/NAP1L1 axis in the regulation of NK cell‐mediated cytotoxicity and chemoresistance, further investigation of this axis using clinical samples and animal experiments is required to confirm and expand these findings.

## 5 CONCLUSIONS

In conclusion, a novel SP2/circPUM1/miR‐770‐5p/NAP1L1 network was identified in OSCC cells, which might be a promising direction for exploring new diagnosis or clinical treatment targets.

## AUTHOR CONTRIBUTIONS

**Lian Liu:** Supervision (equal); validation (equal); visualization (equal); writing – review and editing (equal). **Chen Zou:** Formal analysis (equal); investigation (equal); methodology (equal); software (equal); validation (equal); writing – original draft (equal). **Xiao‐zhi Lv:** Investigation (equal); software (equal); validation (equal). **Haigang Wei:** Software (equal); validation (equal). **Siyuan Wu:** Software (equal); validation (equal); visualization (equal). **Jing Song:** Formal analysis (equal); visualization (equal). **Zhe Tang:** Methodology (equal); validation (equal); visualization (equal). **Hailing LUO:** Data curation (equal); software (equal); validation (equal). **Xia Li:** Conceptualization (equal); investigation (equal); resources (equal); supervision (equal); writing – review and editing (equal). **Yilong Ai:** Conceptualization (equal); data curation (equal); investigation (equal); project administration (equal); supervision (equal); validation (equal); writing – original draft (equal); writing – review and editing (equal).

## CONFLICT OF INTEREST STATEMENT

The authors declare that they have no competing interests.

## Supporting information


Figure S1

## Data Availability

All data that support the findings of this study are available from the corresponding authors upon reasonable request.
