# Supplementary material for: SP2‐induced circPUM1 modulates chemoresistance and nature killer cell toxicity in oral squamous cell carcinoma
Source: J Cell Mol Med. 2023 Aug 9;28(5):e17888. doi: 10.1111/jcmm.17888 (PMC10902577; doi:10.1111/jcmm.17888)
Supplement: Supplementary file 1 — Figure S1 [file JCMM-28-e17888-s001.docx]

Supplementary Figure 1


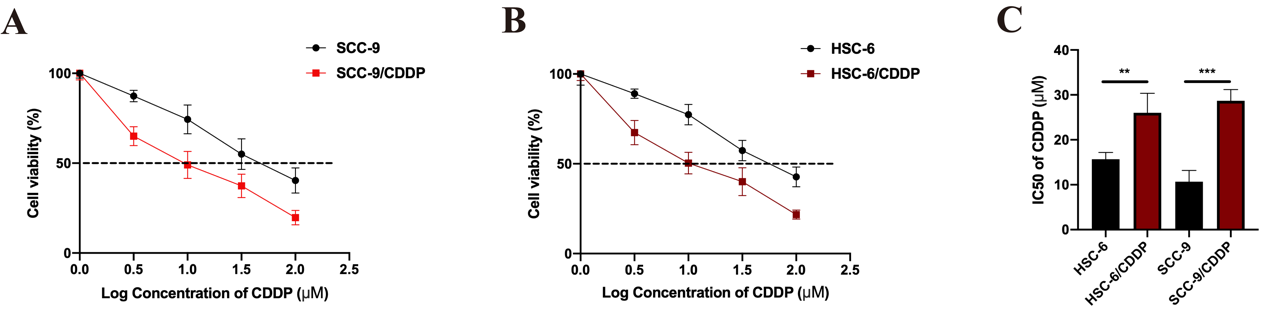


A and B: Cell viability of SCC-9, SCC-9/CDDP, HSC-6, and HSC-6/CDDP cells. C: The half-maximal inhibitory concentration (IC50) of CDDP was determined in the dose response curves.
